# Cr/13X Zeolite and Zn/13X Zeolite Nanocatalysts Used in Pyrolysis of Pretreated Residual Biomass to Produce Bio-Oil with Improved Quality

**DOI:** 10.3390/nano12121960

**Published:** 2022-06-08

**Authors:** Elena David, Adrian Armeanu

**Affiliations:** National Research Institute of Cryogenics & Isotopic Technologies, Street Uzinei No. 4, P.O. Râureni, P.O. Box 7, 240050 Râmnicu Vâlcea, Romania; adrian.armeanu@icsi.ro

**Keywords:** nanocatalysts, 13X zeolite support, corn cobs biomass, acid washing, pyrolysis, bio-oil, hydrocarbons

## Abstract

By loading Cr and Zn on 13X zeolite, efficient nanocatalysts were prepared; they were characterized by different techniques and used for corn cobs pyrolysis to produce bio-oil. The corn cobs biomass (CCB) was washed with sulfuric acid 0.1 M, and the characteristics of the pretreated biomass (PTCCB) were analyzed. Pyrolysis was performed at different catalyst-to-biomass ratios (C/B), and the composition of the obtained bio-oil was determined. The results showed that the crystallinity of the nanocatalysts was slightly lower than that of the pattern 13X zeolite. The surface observation of the nanocatalysts showed the presence of pores and particles, which are quite evenly dispersed on the surface, and no difference was observed in the morphology of the Zn/13X zeolite and Cr /13X zeolite nanocatalysts. In comparison to 13X zeolite, the morphological changes, metal dispersion, and surface area decrease of both Zn/13X and Cr/13X zeolite nanocatalysts could be observed. Pyrolysis tests demonstrated that the use of Zn/13X zeolite and Cr/13X zeolite nanocatalysts could be very profitable to obtain a high conversion to hydrocarbons of the compounds containing oxygen, and consequently, the quality of the bio-oil was improved.

## 1. Introduction

The pyrolysis process is considered a promising way to produce economical renewable fuels and chemicals starting from different sources of biomass [1,2,3]. To date, the major application of pyrolysis that have mainly been studied is bio-oil production, which is considered a second-generation energy carrier [4]. However, the liquid fraction including oil phase consists of a mixture of oxygenated compounds and hydrocarbons [5,6], and the oil phase usually has a high acidity and viscosity, low stability, corrosive nature, and low vapor pressure. All these are the major limitations that make it impossible to use directly as a bio-fuel, as shown in studies [7,8,9,10]. There are two major routes to improve the quality of bio-oil: upgrading the biomass raw materials through pretreatment before pyrolysis [11,12,13] and a catalytic method of deoxygenating the bio-oil created by the pyrolysis process [14,15,16,17,18]. As shown in references [15,19], the biomass washing with a dilute acid could remove over 90% of ash content; therefore, acid washing should have a significantly positive influence on biomass quality. The catalytic processing of pyrolysis vapors is the second way used to improve the quality of the bio-oil [20,21,22,23,24]. 

The catalytic pyrolysis process can be performed in two different ways, depending on how the catalyst is placed in the reactor: in situ, when the catalyst is mixed with the biomass, and ex situ, when the catalyst is located outside the area containing the biomass, experiments of this type being used in a number of studies as well [25,26,27,28,29,30,31,32].

Different nanocatalysts such as zeolites have shown good performances in the deoxygenation reaction of oxygenate compounds from the bio-oil [33,34]. However, other studies [35,36,37] have mentioned that the in situ pyrolysis of biomass causes the formation of a larger amount of coke on the surface of the catalyst, which leads to the deactivation of the catalyst, a phenomenon due to the fact that the catalyst is mixed with biomass and forms a single zone. In addition, because the catalyst is mixed with biomass, its regeneration is more difficult, compared to the ex situ process where the catalyst can be easily removed from the reaction zone of the reactor for regeneration and reuse.

The results from previous studies [34,35,37,38,39,40] have suggested that the deoxygenation reactions of oxygenated compounds from the bio-oil composition can be efficiently performed in the in situ and ex situ processes.

On the other hand, zeolites are also known as being very effective molecular sieve materials. Some examples of zeolites showing sharp sieving properties (acting as molecular sieves) are shown in references [41,42,43]. In addition to being an effective molecular sieve material, Na-13X zeolite also has the ability to retain water and CO_2_ from air and inert gases. Due to its large surface area and defined pore diameter, it can also be used to support catalysts in the biomass conversion processes. Currently, few studies of this have been reported.

Therefore, aiming at the chemical treatment of the biomass by acid washing, Zn/13X zeolite and Cr/13X zeolite nanocatalysts were prepared. Their deoxygenation potential of the oxygenated compounds present in the bio-oil resulting from the pyrolysis processes (in situ, ex situ, or combined) of PTCCB was demonstrated. These two nanocatalysts are different from what is known, for example, comparing the characteristics of ZSM-5 zeolite (one of the most studied and used zeolites in biomass conversion processes) with 13X zeolite characteristics: it was found that 13X zeolite has a bigger specific surface area than ZSM-5, 682 m^2^/g compared to 420 m^2^/g, respectively [44]. In addition, the average pore size of the 13X zeolite is bigger than that corresponding to the ZSM-5 zeolite (1 nm compared to 0.54–0.56 nm, respectively) [45]. Considering these characteristics and that biomass components have bigger molecules, 13X zeolite could be a suitable catalytic support to obtain effective nanocatalysts in the pyrolysis process, favoring bio-oil production and its quality improvement. Thus, the use of acid-treated biomass (using 0.1 M H_2_SO_4_) and these nanocatalysts in a combined process (in situ and ex situ variants) [46] make it possible to produce a bio-oil product with an improved quality.

The rationale for selecting Cr and Zn as doping metals was based on the fact that the main challenge for converting biomass to liquid fuels is in the design of new catalysts to promote the reactions of deoxygenation, cracking, and aromatization with high efficiency and to resist catalytic activity degradation. 

Among the four most active metals, Fe, Co, Ru, and Ni [47,48,49], Fe- or Co-doped catalysts have been found to form clusters quite easily by sintering, destabilizing the surface and reducing the catalytic efficiency. Various studies [50,51,52] have shown that zinc, if added as a promoter to iron-based catalysts, prevents iron cluster formations from sintering and stabilizes the surface area of iron oxide, which led to the idea that this metal could have a good behavior and activity in the process of the pyrolysis of biomass. Additionally, Zn/ZSM-5 has been reported to be an excellent aromatization catalyst with, in some cases, a performance superior to that of Ga/ZSM-5 [53,54], and bimetallic Zn/ZSM-5 catalysts have been reported to show increased aromatics selectivity and increased stability compared to the parent Zn/ZSM-5 [55,56].

Despite a large number of studies concerning noble metals, their use is not ideal, owing to some restrictions. First, the price of these metals may limit their use. Secondly, it has been established that they are not resistant to severe thermal treatment: the sintering of metal particles is associated with a loss of catalytic activity. Thus, new active phases less sensitive to high temperature in the presence of water are desirable. In this sense, transition metals appear to be promising candidates. Chromium metal is notable for its high corrosion resistance and hardness, as evidenced by many studies [57,58,59]. Therefore, in the present work, chromium was chosen as a doping metal in the catalyst design. 

## 2. Materials and Methods

### 2.1. Corn Cobs Biomass

**Figure 1:**
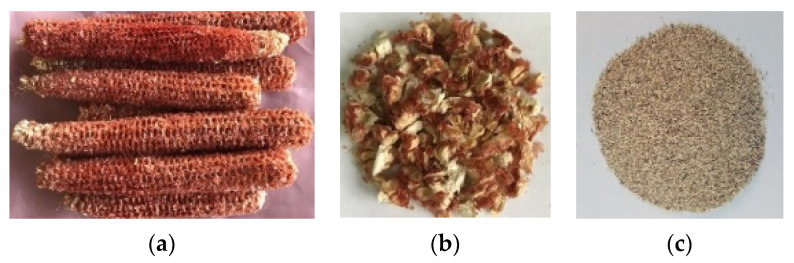
Images of corn cobs biomass (CCB) (**a**) as received; (**b**) crushed; (**c**) sieved (≤0.25 mm).

In this study, corn cob biomass (CCB), harvested from the local area, were crushed and sieved by 60 mesh sieve and then were air-dried in the oven at 110 °C for 3 h (Figure 1a–c). The CCB used was a fraction of ≤60 mesh (0.25 mm) to minimize the heating and mass transfer impact and to reduce the temperature gradients into the CCB sample. A sulfuric acid (0.1 M H_2_SO_4_) solution was used to wash the biomass. Then, 100 g of CCB was immersed in an Erlenmeyer flask containing 500 mL of H_2_SO_4_ (0.1 M) and maintained at room temperature (~22 °C) for 6 h.

The washed CCB was filtered through a filter funnel, the separated solid was washed with distilled water until the filtrate was neutral to pH and was dried at 110 °C for 4 h, and the resulting material was labeled as pretreated corn cob biomass (PTCCB). 

### 2.2. Corn Cob Biomass Analysis

CCB proximate analysis was made according to ASTM standards D 2016 74, D3174 89, and D1102 84 to determine the fixed carbon, moisture, volatile and ash content. The final analysis was performed according to ASTM D 5373 to determine the content of carbon, hydrogen, nitrogen, sulfur, and oxygen using a Carlo Elba 1106 instrument. The composition was established according to ASTM D 3176, and using ASTM D240-02 standard, the gross calorific value (GCV) was determined. The extractives and lignocellulosic were determined using TAPPI test method, as is described in reference [60]. 

The metals content was determined by atomic absorption analysis using a spectrophotometer type Analytic Yena Nova 300. The Organic structure of CCB and PTCCB was obtained through Fourier infrared spectroscopy using a FTIR spectrometer Nicolet iS50. CCB and PTCCB were subjected to thermogravimetric analysis using a Setaram Setsys Evolution instrument coupled with a PC under N_2_ of 99.99% purity, at a flow rate of 50 mL/min, and with a heating rate of 20 °C/min up to a final temperature of 700 °C. 

In all tests, the sample mass was of 50 mg, and each analysis/test was repeated three times. The reported values represent the average of these three tests, the relative error was less than 1.5%, and the data standard deviations are provided. 

### 2.3. Preparation and Characterization of Nanocatalysts

**Figure 2:**
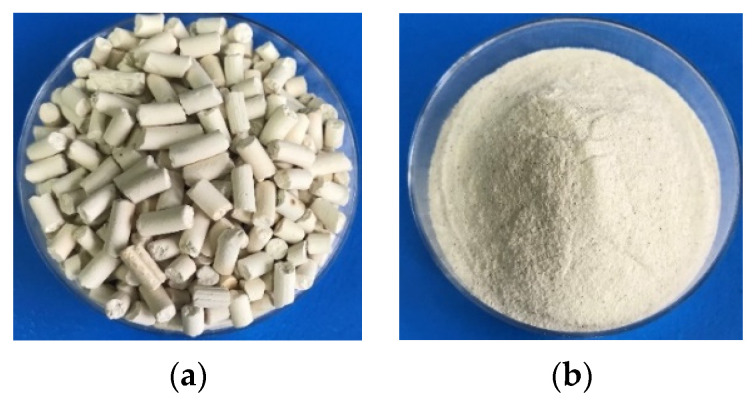
Images of: (**a**) 13X zeolite in pellets form; (**b**) powder of 13X zeolite.

A commercial zeolite type 13X, in pellets form (Ꝋ = 3 mm), with a Si/Al ratio = 3.2, was purchased from FLUKA of Sigma-Aldrich Holding AG (Figure 2). The zeolite pellets were milled, sieved to particles with a size ≤ 0.5 mm, and calcined at 500 °C for 3 h. According to previous studies [61,62,63], a metal loading value in the range of 8 to 10 wt.% in the catalyst is necessary to obtain a sufficient number of strong catalytic active sites for deoxygenation reactions. A higher metal content reduces the number of these active sites and inhibits the catalytic activity. In the work, the nanocatalysts containing a metal load around 9 wt.% were prepared by the wet impregnation method. 

To prepare 50 g of Zn/13X zeolite or Cr/13X zeolite, 20.47 g Zn(NO_3_)_2_·6H_2_O or 20.60 g Cr(NO_3_)_3_·9H_2_O was dissolved in 75 mL of distilled water, and then 45.5 g of 13X zeolite was added in the solution and mixed for 8 h under constant magnetic stirring. To evaporate the water, the mixture was heated at 85 °C for 2 h and dried in an oven at 110 °C for 8 h. The resulting material was further calcined at 500 °C for 6 h to produce the final nanocatalyst. The atomic absorption analysis results showed that 8.85 wt.% of Zn and 9.72 wt.% of Cr were present in Zn/13X and Cr/13X zeolite nanocatalysts, respectively.

The prepared Zn/13X zeolite and Cr/13X zeolite nanocatalysts were characterized by XDR, using D/max-2200/PC, Rigaku, Japan, and copper KR radiation (40 kV, 20 mA) as the X-ray source. The size of metallic crystallite was determined based on Scherrer Formula (1):

d_crystallite_ = 0.94λ/B·cos θ (1)

where d_crystallite_ size is determined in nm, B is full width at half maximum of the most intense peak from spectrum (FWHM), λ is considered 1.5405 Å, and 2θ angle is in the range 10 to 90. Using Quantachrome Inst., Nova 2200e analyzer, the N_2_ adsorption/desorption isotherms at (−196.15 °C) were determined, and the Brunauer–Emmett–Teller method was employed to determine the specific surface area of catalysts.

The total pore volume (V_tp_) was established from the N_2_ quantity adsorbed for a relative pressure (P/P_O_) of 0.99. The nanocatalyst morphology was analyzed by scanning electron microscopy (SEM), using a microscope JSM-7500 F (JOEL-Japan) operated at 10 kV, and gold coating was used. The nanocatalyst acidity was analyzed by the ammonia temperature programmed desorption method (NH_3_-TPD). For the TPD-NH_3_ measurements, a Micrometrics Autochem 2920 instrument equipped with a thermal conductivity detector (TCD) was used.

### 2.4. Pyrolysis Experiments

**Figure 3:**
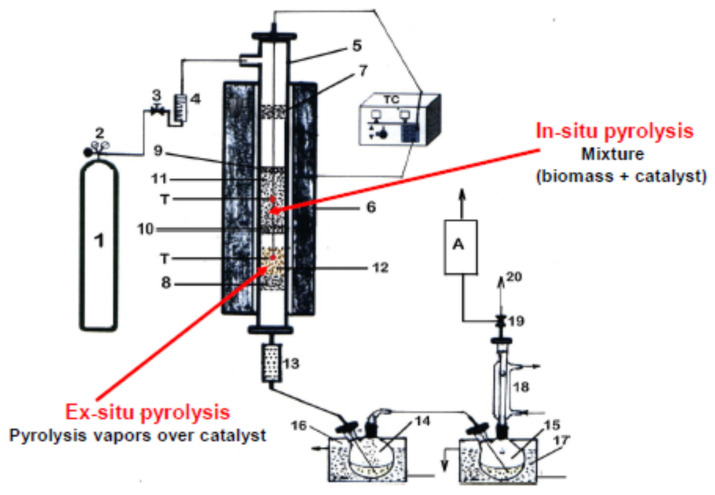
The scheme of experimental system used for biomass in situ, ex situ and combined pyrolysis process. (1)—tank of N_2_ gas under pressure; (2)—pressure gauge; (3)—control valve; (4)—flowmeter; (5)—reactor; (6)—electrical oven; (T)—thermocouple for temperature control; (7), (8) and (10)—perforated metal support; (9)—glass wool pad; (11)—biomass bed/mixture (biomass and catalyst) bed; (12)—catalyst bed; (13)—ceramic filter; (14) and (15)—gas–liquid separator with liquid product; (16)—cooling water bath (5 °C); (17)—cooling ice bath (0 °C); (18)—condenser with water (5 °C); (19)—valve with two way; (20)—gaseous product. (A)—analyzer.

All pyrolysis experiments were carried out under an N_2_ atmosphere in a fixed bed reactor (length of 450 mm, inner diameter of 12 mm) made of stainless steel. A scheme of the experimental system is shown in Figure 3. To ensure an isothermal regime, the reactor was heated by an electrical oven. The experiments were performed at 500 °C, with 50 °C/min heating rate and a N_2_ gas flow rate of 60 mL/min, and for each experiment, 20 g of PTCCB with particle size ≤0.25 mm were used. For pyrolysis tests, the nanocatalyst was mixed with PTCCB in different ratios, as is shown in Table 1. 

During in situ tests, the area corresponding to the ex situ process was kept empty. After the series of in situ and ex situ tests were performed with each type of catalyst, the obtained results were compared, and the catalysts with the best performance in in situ and ex situ tests were chosen. These catalysts were further evaluated in the combined pyrolysis process.

In the ex situ pyrolysis experiments, the catalyst bed was placed after the biomass bed. In the combined tests, Zn/13X zeolite was employed in the in situ pyrolysis process, and Cr/13X zeolite was employed in the ex situ pyrolysis process. 

The particle size of catalysts used in all experiments was ≤0.5 mm. Due to the way biomass and catalyst are placed in the reactor, in the ex situ experiment, the thermal pyrolysis of the biomass took place first, and then the released vapors (primary product) passed over the catalyst bed. The resulting vapors were then passed through a metallic sieve and ceramic filter to retain any solid particles and through traps to collect the liquid phase.

The bio-oil contained in the liquid fraction was separated by dissolving in dichloromethane (DCM) and then filtered over glass wool and calcium chloride. The bio-oil mass was determined by weighing, the solid phase consisting of bio-char, and nanocatalyst together with the coke deposited on and into nanocatalyst mass were also weighed to determine the pyrolysis yield for this product. Each pyrolysis test was carried out at least three times; an average of these values was considered as the final result, and the measurement error was less than 0.5%. The bio-oil composition was identified using GCMS-QP2010SE Gas Chromatograph/Mass Spectrometer, and for the identification of the chromatographic peaks the NIST mass spectrum library was used. 

The detected compounds were classified into 9 main groups: aliphatic hydrocarbons, aromatic hydrocarbons, acids, aldehydes, ketones, phenols, furans, nitrogen compounds, and other compounds (compounds containing halogen, sulfur and silicon).

### 2.5. Coke Deposition on the Nanocatalysts 

To analyze the coke formation and its deposition on catalysts after the pyrolysis process, a thermogravimetric device type TGA/DSC Stare, from Mettler Toledo, Ltd. was used. Oxidation-based analysis at the programmed temperature (TPO) was performed for spent catalyst, and the obtained results were compared with the fresh catalyst. 

A quantity of 20 mg catalyst was heated from 20 °C (~room temperature) to a temperature of 900 °C with a heating rate of 10 °C/min, in an atmosphere of air at a flow rate of 80 mL/min, and nitrogen gas at a flow rate of 15 mL/min, respectively. The coke amount was established as a percentage calculated by the difference between the mass of the initial sample and the mass of the residual sample. 

## 3. Results and Discussion

### 3.1. Influence of Acid Washing on Biomass Characteristics

The results obtained by proximate, ultimate, and components of CCB and PTCCB samples analysis are presented in Table 2. 

The Results showed, after acid washing, that the moisture, fixed carbon, and ash contents in CCB decreased from 8.27 wt.%, 12.22 wt.%, and 3.46 wt.% to 6.14 wt.%, 11.19 wt.%, and 2.15 wt.%, respectively, while the volatiles content increased from 76.05 wt.% to 80.52 wt.%. The moisture content decreasing in the biomass particle voids makes them more effective in volatiles release, as is shown in the study in reference [64] and the increase in the volatiles content and the decrease in the ash amount in the biomass determine an improvement of the heating value, as is presented in the reference [65]. After acid washing, the carbon content of PTCCB increased by 9.26 wt.%, while the oxygen content decreased by 11.16 wt.%. The O/C molar ratio was reduced by 18.51%, showing that acid washing had a good biomass deoxygenation effect, this result being similar with data presented in other studies [66,67,68].

As can be seen in Table 2, the cellulose, hemicellulose, and lignin content of PTCCB increased, which means that a significant portion of extractives by acid washing was removed, and this change could have an important influence on the pyrolysis process and product quantity.

The contents of metals from CCB and PTCCB were determined (Table 3). The results showed that the contents of Na, K, Ca, Mg, Fe, Al, Cu, and Zn decreased after washing with H_2_SO_4_. The removal rate was in the range 72% to 98.46%. The highest removal rate was for Ca (98.46%), and the lowest rate was for Al (72%). These results show that acid washing could effectively remove the metals from CCB and PTCCB.

According to other studies [69,70], Ca and Mg in biomass exist not only in form of carbonate, but also in other forms such as silicate, phosphate or other compounds, which are less soluble in organic acid. To remove these metals, 0.1 M sulfuric acid solution has been proven to be quite effective. 

It is also known from other studies [21,69,70] that alkaline and alkaline earth metals play an important role in biomass conversion (pyrolysis, gasification, combustion) for corrosion and deposition at high temperature; therefore, their removal is beneficial. Other metals (Cu, Fe, Zn) can exist in biomass in various forms through bonds created to biomass polymers containing oxygen functional groups, such as carboxylic, carbonyl or phenolic groups in cellulose and lignin [19,70]. Changing the metals content in biomass has an influence on catalytic cracking reactions, and consequently, the bio-oil characteristics are affected, and the products distribution is modified; as the metals content decreases, the bio-oil yield increases, and the char yield decreases [68]. Taking into account these findings, in all the in situ, ex situ and combined pyrolysis tests, biomass samples pretreated with sulfuric acid (PTCCB) were used (as described in Section 2.1). 

### 3.2. Thermal Degradation Analysis

**Figure 4:**
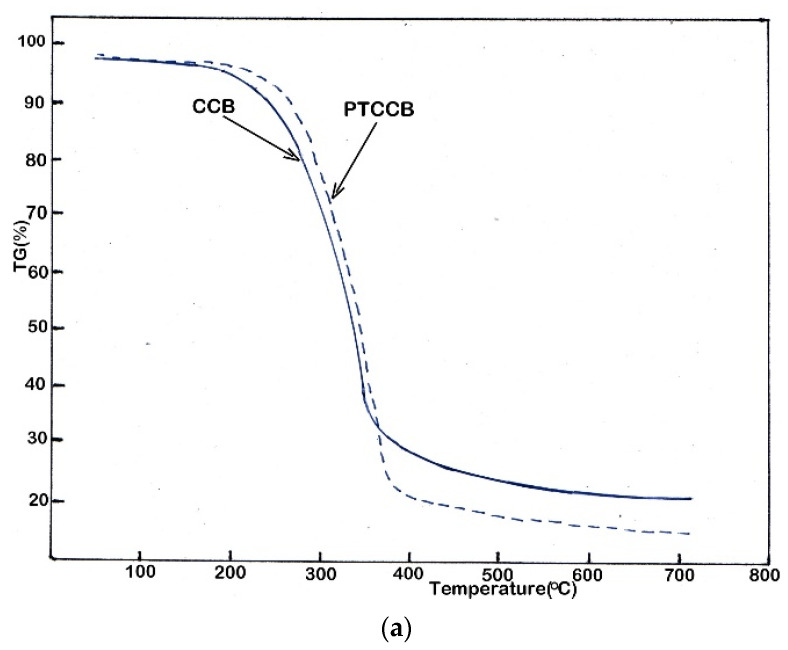

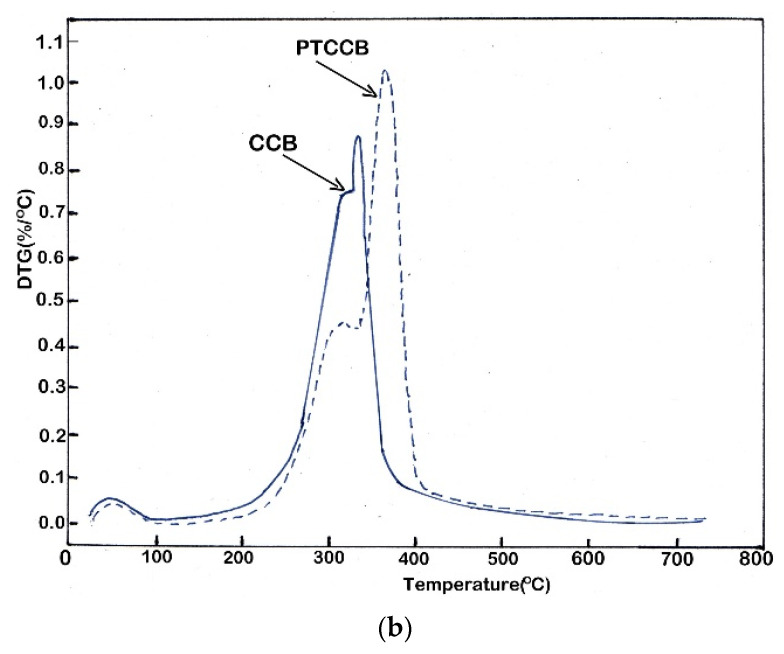
The profile of TG (**a**) and DTG (**b**) curves obtained for CCB and PTCCB samples.

TG and DTC curves of CCB and PTCCB obtained with a heating rate of 20 °C/min up to a final temperature of 700 °C are shown in Figure 4. The process of the decomposition of CCB and PTCCB samples passed through three steps. In the first stage, which took place at a temperature below 200 °C, a small weight loss was obtained due to the evaporation of moisture and a vitrification process. The second step took place in the temperature range of 200 °C to 400 °C and was the main decomposition stage, during which the CCB and PTCCB samples lost about 68.22% and 78.95% weight, respectively, a loss that was due to the severe decomposition of cellulose and hemicellulose and lower decomposition of lignin, this being shown in other studies [71,72,73]. The third step took place above the temperature of 400 °C and consisted of the decomposition of the remaining lignin and of a carbonization process, with a slow weight loss. For DTG curves, a small difference between the CCB and PTCCB samples was observed in the first and the third steps, while in the second step of the DTG curves, two peaks were obtained. The first peak was due to the decomposition of hemicellulose, and the second peak was due to the rapid cellulose decomposition. The catalytic effects of AAEM could reduce the activation energy and cause a decrease in the decomposition temperature [74].

Therefore, in the PTCCB sample in which the AAEM content decreased by acid washing (Table 3), the DTG curves shifted to higher temperature and became wider in the second stage. The DTG curve for PTCCB presents a lower shoulder peak, which can be attributed to the removal of a hemicellulose amount in the acid pretreatment.

However, the second peak of PTCCB is higher than that of CCB and is clearly due to the destruction of the cellulose structure by acid washing and to decomposition at an increased temperature.

### 3.3. Nanocatalyst Characterization

As was shown, the 13X zeolite was chosen because it has a bigger specific surface area and a bigger average pore diameter than that corresponding to the ZSM-5 zeolite. Taking into account that biomass intermediates have bigger molecule size, 13X zeolite could be a suitable support material for catalysts and would help to improve the bio-oil production and its quality by increasing the hydrocarbons content. The physical and chemical characteristics of the catalysts that were prepared were determined by different techniques, as described in Section 2.3. 

**Figure 5:**
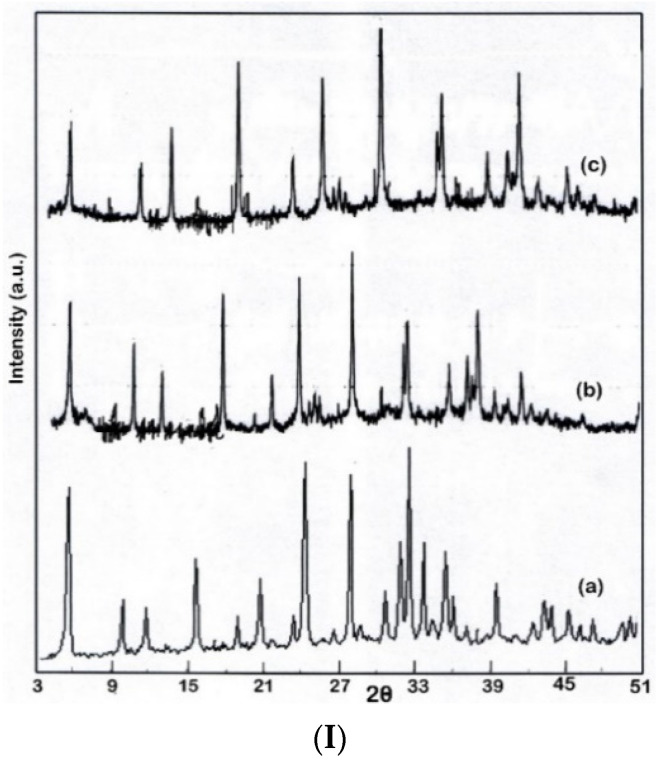

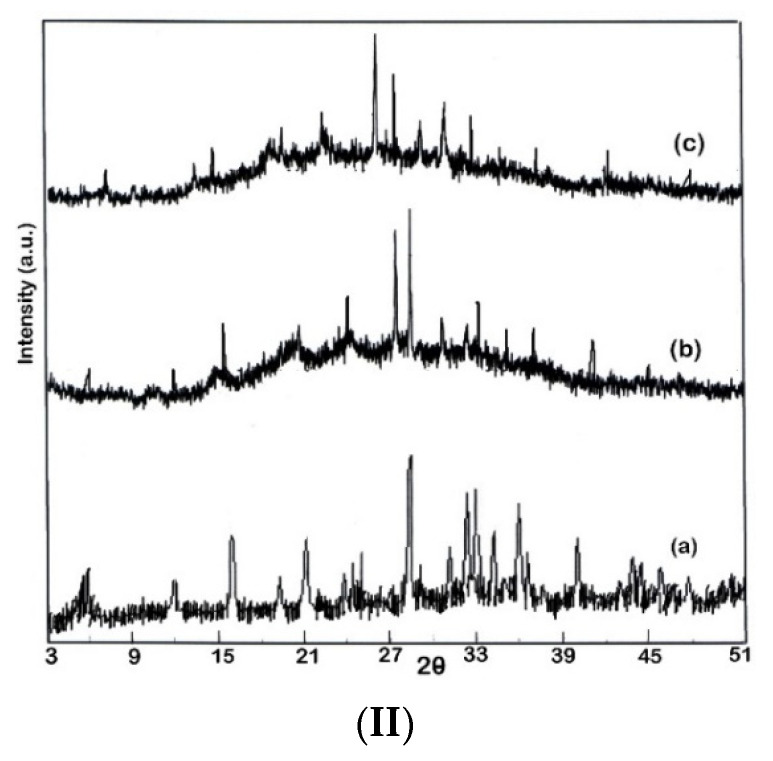
The XRD measurements of: (**I**) fresh 13X zeolite (a); fresh Zn/13X zeolite (b) and fresh Cr/13X zeolite (c); (**II**) spent 13X zeolite (a); spent Zn/13X zeolite (b) and spent Cr/13X zeolite (c).

Figure 5 shows the XRD measurements of fresh 13X zeolite, fresh Zn/13X zeolite and fresh Cr/ 13X zeolite (I), and spent catalysts (II) (13X zeolite, Zn/13X zeolite, and Cr/ 13X zeolite) recovered after the pyrolysis process. 

**Figure 6:**
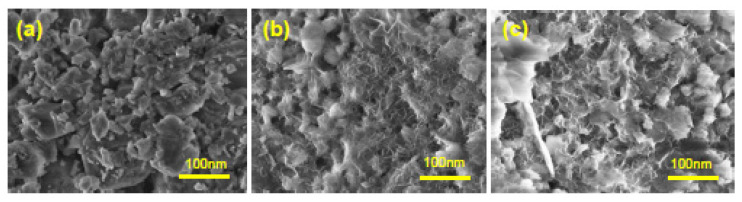
SEM images of: (**a**) fresh 13X zeolite; (**b**) fresh Cr/13X zeolite; (**c**) fresh Zn/13X zeolite.

As can be seen in Figure 5, the fresh 13X zeolite presents a higher crystallinity than that of the fresh Zn/13X and Cr/13X catalysts. The XRD patterns of the fresh 13X zeolite shows the typical peaks corresponding to faujasite (FAU) zeolite structure. The catalyst samples (fresh Zn/13X zeolite and fresh Cr/13X zeolite) show very similar patterns to the fresh 13X zeolite; however, the crystallinity indicated by the peak intensity is slightly decreased. In contrast, the spent catalysts (13X zeolite, Zn/13X zeolite and Cr/13X zeolite) still show low diffraction peaks in intensity, which can be attributed to the presence of the crystalline forms of Zn and Cr, but in much smaller quantities. These results are in agreement with other studies [34,37]. In Figure 6 are shown the SEM images for fresh 13X zeolite, Zn/13X zeolite and Cr/ 13X zeolite catalysts. 

The surface observation of the Zn/13X zeolite and Cr/3X zeolite samples (Figure 6b,c) show the presence of pores and particles, which are quite evenly dispersed on the surface. 

In addition, no difference is observed in the morphology of the Zn/13X zeolite and Cr /13X zeolite samples. In comparison to 13X zeolite, the morphological changes of the Zn/13X and Cr/13X catalysts can be observed, attesting that the metal particles were well-dispersed on the surface of the support. Table 4 presents the specific surface area and porous characteristics for 13X zeolite, Zn/13X zeolite, and Cr/13X zeolite catalysts. 13X zeolite exhibits a surface area of 682 m^2^/g, and the addition of Cr and Zn reduced the specific surface area. Cr/13X zeolite catalyst presented a surface area of 286 m^2^/g, which is 58.05% lower than that for 13X zeolite but 23.77% higher than the Zn/13X zeolite. The Zn/13X zeolite had a smallest surface area of 218 m^2^/g. 

This decreasing is due to the metal ions incorporation on the 13X zeolite support or into the cavities from the zeolite structure. V_tp_ was lower in Cr/13X zeolite and Zn/13X zeolite than that in 13X zeolite, which exhibits a pore volume of 0.678 cm^3^/g, compared to 0.546 cm^3^/g and 0.507 cm^3^/g, respectively. The reduction in V_tp_ in Cr/13X zeolite and Zn/13X zeolite samples indicates the blockage of pores, which is attributed to the loading of the metals into the zeolite structure [3,4,13]. The acidic properties of 13X zeolite and zeolite-based catalysts were evaluated using the NH_3_-TPD method. In Cr/13X zeolite and Zn/13X zeolite catalysts, the acidity increased, and this is due to the presence of a proton that has an acidic character. The total acidity data are presented in Table 5. It can be observed that 13X zeolite exhibited a total acidity amount of 62 μmol/g, while Cr/13X zeolite and Zn/13X zeolite presented total acidity amounts of 91 μmol/g and 72 μmol/g, respectively. 

**Figure 7:**
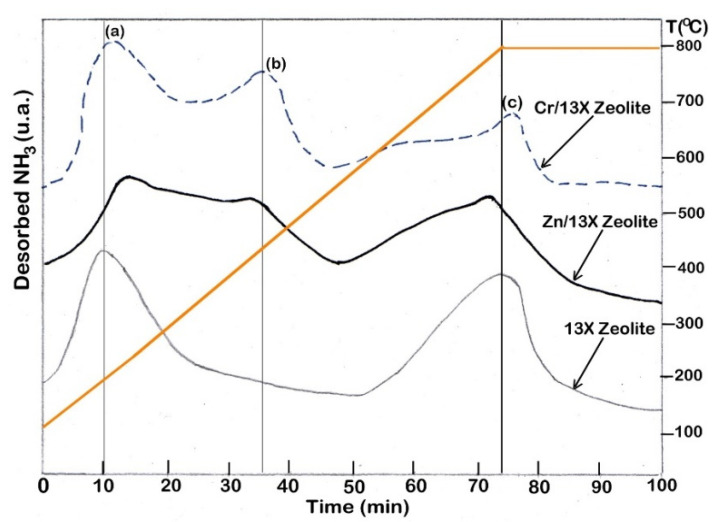
The profile of NH_3_-TPD curves for 13X zeolite, Cr/13X zeolite and Zn/13X zeolite. The peak (a) is characteristic to 13X zeolite and it also appeared for Zn/13X zeolite and Cr/13X zeolite catalysts; The loading of Zn and Cr on the 13X zeolite created new peaks in the spectrum (b and c).

In addition, in Figure 7, it can be observed that loading of Cr and Zn determined the appearance of the new peaks (b and c), which could be due to the creation of new sites with acidic character on the support. This result is supported by previous studies [4,13,30,38] that have shown that the dispersion of metals on support modifies the acid character of catalysts by reducing the number of Brönsted sites but increasing the number of Lewis sites. According to these findings, the increase in total acidity resulted from the increase in the number of Lewis acid sites [30,38]. The decrease in the number of Brönsted acid sites may be related to the impregnation process of the metal precursor. Some hydrogen ions (H^+^) present in the zeolitic support were exchanged by Cr or Zn ions during the impregnation procedure.

Based on these results, it can be concluded that the loading of metals (Cr or Zn) in zeolite has led to an increase in the total acidity, but the number of Brönsted acid states decreased while the number of Lewis acid states increased. 

### 3.4. In Situ Pyrolysis of Corn Cobs Biomass

**Figure 8:**
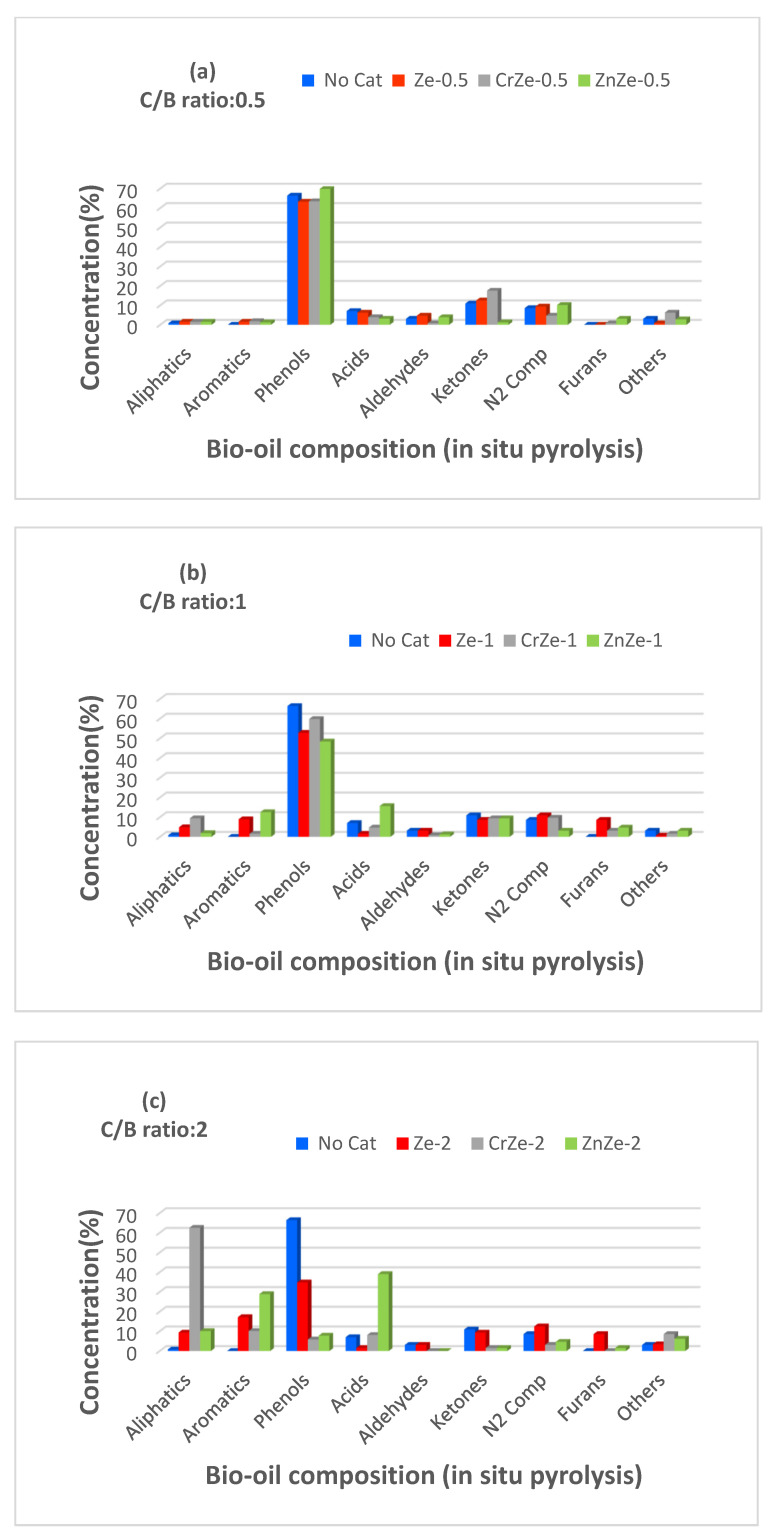
Bio-oil composition resulted by in situ pyrolysis of PTCCB; (**a**)—catalyst-to-biomass ratio was 0.5; (**b**)—catalyst-to-biomass ratio was 1; (**c**)—catalyst-to-biomass ratio was 2 (see Table 1).

Figure 8 shows the bio-oil composition produced from in situ pyrolysis with catalysts and without catalysts of PTCCB. It can be observed that pyrolysis without catalysts produced a bio-oil that is rich in oxygenated compounds (about 66.41% phenols, 7.03% acids, 10.94% ketones).

The high proportion of phenols resulted from lignin pyrolysis, while the acid products and ketones could be especially due to the thermic decomposition of cellulose and hemicellulose compounds from biomass composition. 

The catalysts addition to PTCCB and then in situ pyrolysis pointed out an important decreasing in the oxygenated compounds formation. It was also observed that the using of 13X zeolite without metal loading in PTCCB pyrolysis has a good activity in deoxygenation reactions (Ze_1_ and Ze_2_ decreased the phenol amount to 52.82% and 34.81%, respectively).

On the other hand, the ratio of ketones to acids in bio-oil composition has increased for the biomass samples that were subjected to the pyrolysis process in the presence of catalysts (i.e., to 4.48 for Cr/Ze 0.5 and 3.25 for Zn/Ze 0.5) compared with the pyrolysis process without catalysts of 1.55 ratio. The addition of Cr and Zn on the 13X zeolite support produced catalysts that showed good activity for deoxygenation reactions, and the amount of hydrocarbons in the bio-oil samples considerably increased (Figure 8b,c). From the in situ pyrolysis of the catalyst/biomass mixture in a ratio of 2/1, the bio-oil with the lowest content of oxygenated compounds and a maximum content of hydrocarbons was obtained for the Cr/13X zeolite (Figure 8c), the bio-oil containing 62.53% aliphatics and 10.16% aromatics hydrocarbons, respectively, and a total of 15.58% oxygenated compounds compared to a content of 87.11% oxygenated compounds present in the bio-oil obtained without catalyst (Figure 8c). This can be explained by the fact that a larger amount of catalyst contains a higher number of active states that react with pyrolysis vapors, resulting in the deoxygenation rate enhancing and an increase in hydrocarbon production compared to C/B lower ratios. It should be noted that ZnZe-2 produced 7.81% of phenols and 1.56% of ketones, and CrZe-2 produced 5.79% of phenols and 1.53% of ketones, while in both cases, aldehydes were not be obtained. It also observed that Zn/13X zeolite and Cr/13X zeolite favored acid production, and the acid amount was higher with the increase in C/B ratio. For instance, using ZnZe-0.5 catalyst, 3.12% acid compounds was obtained in the bio-oil, and this percent increased to 39.06% for ZnZe-2. For the CrZe-0.5 catalyst, the bio-oil contained 3.91% acids, and it increased to 8.13% when CrZe-2 was used. These results could be due to the modifications in the pores structure and acid characteristics of the catalyst based on 13X zeolite. The acid states that are present at the outside surface of zeolite and at the pores on its walls take part in deoxygenation reactions. Therefore, poor access to the acidic states present in the structure of the catalysts during in situ pyrolysis leads to the production of oxygenated compounds, such as acids, phenols, and ketones. In addition, the better deoxygenation performance of Cr/13X zeolite compared to Zn/13Xzeolite could be due to its higher total acid character, porosity, and bigger surface area, as can be seen in Table 4 and Table 5. 

### 3.5. Ex Situ Pyrolysis of Corn Cobs Biomass

**Figure 9:**
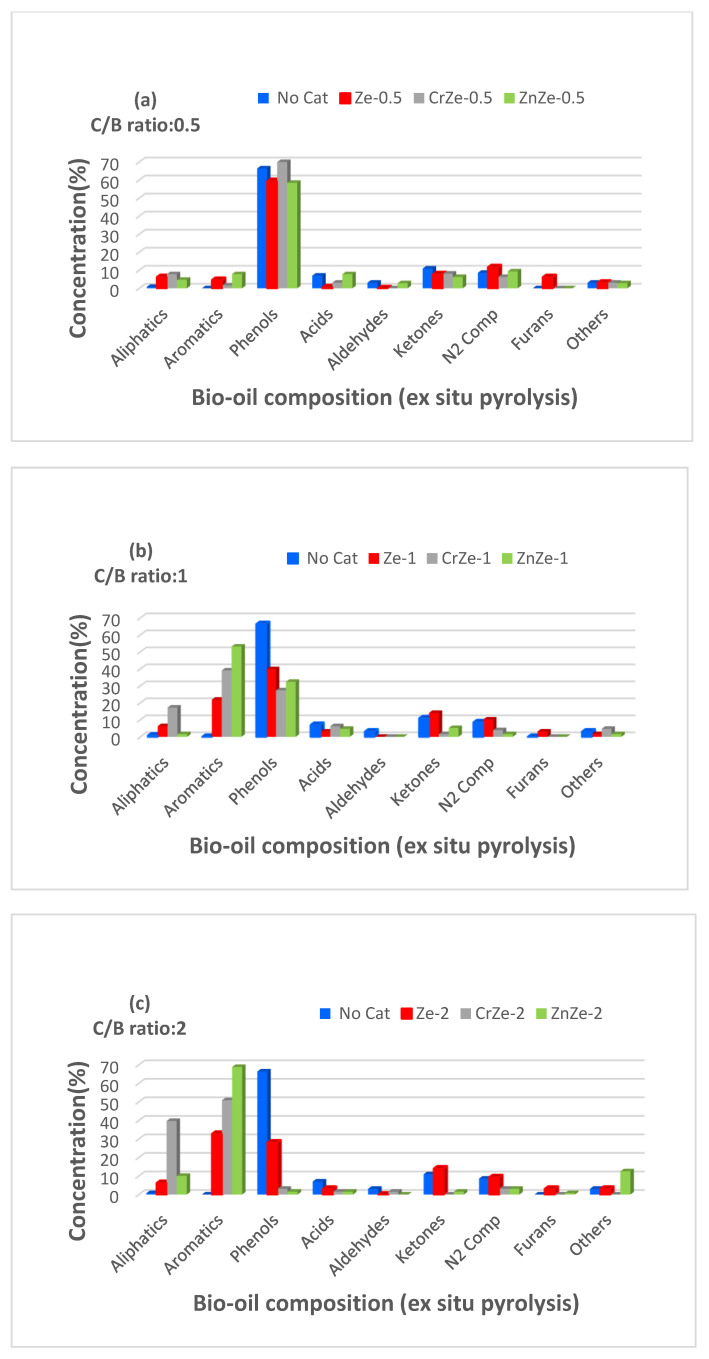
Bio-oil composition resulted by ex situ pyrolysis of PTCCB; (**a**)—catalyst-to-biomass ratio was 0.5; (**b**)—catalyst-to-biomass ratio was 1; (**c**)—catalyst-to-biomass ratio was 2 (see Table 1).

Figure 9 shows the bio-oil composition obtained from the ex situ pyrolysis process of the PTCCB. The obtained results show the different behavior of the catalysts in the ex situ process compared to the in situ process: in the first variant, a higher conversion of the oxygenated compounds was obtained than in the in situ process. 

For the ex situ pyrolysis mode, the catalyst/biomass ratio equal to 2 gave the best results; a maximum conversion of the oxygenated compounds was obtained for all the catalysts tested. As can be seen in Figure 9a–c, all catalysts caused a substantial decrease in the amount of oxygen-containing compounds, and this decrease was noticed with the increase in the C/B ratio. Visibly, using ZnZe-2 catalyst, only 1.56% of phenol compounds and 1.53% of ketones were produced.

An important observation was that Zn/13X zeolite catalysts favored the yield of acids during the in situ process, but in the ex situ process, the quantity of acids decreased, being much lower (Figure 8 and Figure 9). For example, in ex situ pyrolysis, using ZnZe-2 catalyst, a bio-oil containing 1.56% acids was obtained, while the bio-oil obtained by non-catalytic pyrolysis contained 7.03% acids, and using ZnZe-2 catalyst in the in situ pyrolysis mode resulted in approximately 39.06% acids in the bio-oil produced (Figure 8c and Figure 9c). Similarly, Cr/13X zeolite catalysts presented an increased activity in acids conversion into non-oxygenated compounds during the ex situ pyrolysis mode; on the other hand, it was observed that the acids amount increased in the in situ pyrolysis. Similar results are presented in other studies [27,30]. Another finding refers to the quantity of aldehydes and ketones determined in the bio-oil composition. At a C/B ratio of 1 or 2 in the bio-oil resulting from the ex situ process, the percentage of aldehydes and ketones was low or zero (Figure 9b,c), as opposed to in situ pyrolysis, where this percentage was higher (Figure 8b,c). These results showed that the catalysts favored decarboxylation and decarbonylation reactions as the favorite conversion reactions in ex situ pyrolysis. The yield of liquid product was 35.8 wt.% for the in situ pyrolysis process and 34.7 wt% for the ex situ pyrolysis process, respectively, and the yield of the components from the bio-oil is related to this liquid fraction. 

### 3.6. Combined In Situ and Ex Situ Pyrolysis of Corn Cobs Biomass

**Figure 10:**
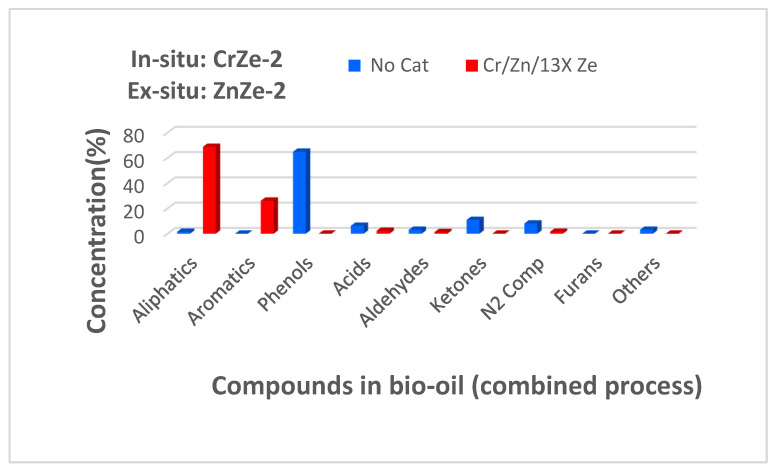
Bio-oil composition resulted by combined in situ and ex situ pyrolysis of PTCCB; catalyst-to-biomass ratio was 2 (see Table 1).

Based on the results presented in Section 3.3 and Section 3.4, the combined in situ and ex situ pyrolysis of PTCCB was tested. Figure 10 shows the results obtained in terms of the composition of the obtained bio-oil. 

The results obtained in the case of combined pyrolysis indicated that the Zn/13X zeolite and Cr/13X zeolite favored the yield of aliphatic and aromatic hydrocarbons. Additionally, the Cr/13X zeolite used in the in situ process favored the yield of aliphatic hydrocarbons and the Zn/13X zeolite employed in ex situ pyrolysis favored the production of aromatic hydrocarbons (Figure 8c and Figure 9c).

Therefore, in order to produce a bio-oil with improved quality containing a higher quantity of aliphatic and aromatic fractions, in the combined process, CrZe-2 catalyst was employed for the in situ sequence, and ZnZe-2 catalyst was used in the ex situ sequence. The results obtained regarding the bio-oil composition show that using a process based on both in situ and ex situ pyrolysis is a better option than the separate use of either the in situ or ex situ pyrolysis process. The C/B ratio equal to 2 led to a higher efficiency in terms of deoxygenation reactions. Table 6 shows the main chemical compounds that have been identified in the bio-oil produced by the combined pyrolysis variant of PTCCB. A bio-oil was obtained containing fewer oxygenated compounds but with a higher hydrocarbons content (Figure 10). In the pyrolysis process based on both in situ and ex situ variants, it is very likely that oxygen-containing compounds that were not converted to hydrocarbons during in situ pyrolysis were successfully thermally transformed into other compounds with lower oxygen content [30]. Moreover, it can also be considered that in situ catalytic pyrolysis has favored the production of phenols, aldehydes, ketones, and even acids, as can be seen by analyzing the results presented in Section 3.3. These chemical compounds containing carbonyl and carboxyl groups passed over the ex situ catalytic bed, and there, they converted mainly by decarbonylation and decarboxylation reactions into various hydrocarbons [34]. These improved deoxygenation reactions that occurred during the combined pyrolysis process and this behavior are due to both the modified texture and the improved acidic properties of the zeolite catalysts (Cr/13X and Zn/13X ), and these characteristics favored the transformation of oxygen-containing compounds into aliphatic or aromatic hydrocarbons.

### 3.7. Evaluation of Coke Deposition on Catalysts 

**Figure 11:**
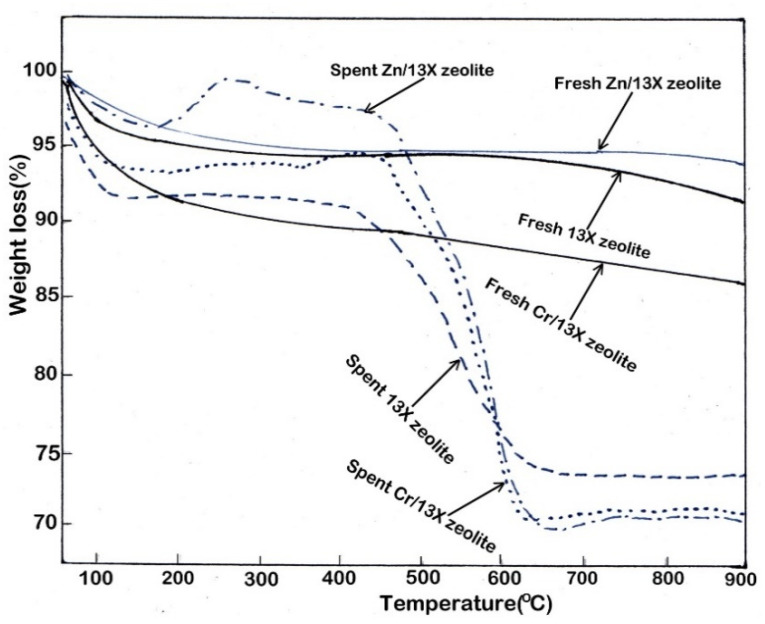
Weight loss of fresh catalysts and spent catalysts, rate of heating of 10 °C/min, in air atmosphere, flow rate of 100 mL/min.

Figure 11 presents the results of the TPO analysis for catalysts, before and after use (fresh/spent catalyst).

The coke deposition on catalysts is attributed to the production of hydrocarbons, mainly aromatic hydrocarbons. It has also been observed that a higher acidic character of the catalysts determined the formation of a bigger coke amount, which, by deposition, contributed to the catalyst deactivation [28]. 

The experimental data obtained show that the carbon amount resulted and deposited on the 13X zeolite was slightly higher than the Zn/13X zeolite catalyst (8.65% compared to 6.43%, respectively), while on the Cr/13X zeolite catalyst, a coke quantity of 14.05% by weight was deposited. The deposition of coke on all catalysts is due to the formation of different types of hydrocarbons on their surface [29]. For spent Zn/13X zeolite and Cr/13X zeolite catalysts, a slight increase in weight was observed compared to fresh catalysts, an increase that can be due to the oxidation reactions of Zn and Cr metallic particles to oxides during the pyrolysis process [61,62]. In general, it can be said that these catalysts have been shown to be effective in deoxygenating the bio-oil, but they can be deactivated by depositing coke on their surface.

## 4. Conclusions

This study showed that washing with sulfuric acid improved the quality of corn cob biomass. The content of cellulose and hemicellulose from biomass composition influences the process of pyrolysis and the quality of resulted products, and an increased content obtained means that an important part of extractives by acid washing was removed. This change had a favorable influence on the pyrolysis process and bio-oil quantity and quality. Zeolite 13X, having a larger specific surface area and an average pore diameter larger than that corresponding to the ZSM-5 zeolite, proved to be a suitable catalytic support, itself having a catalytic activity.

By loading metals (Cr/Zn) on 13X zeolite, efficient catalysts for the pyrolysis process of biomass to produce bio-oil were obtained. The Cr/13X zeolite catalyst used in in situ pyrolysis favored the production of aliphatic hydrocarbons, while the Zn/13X zeolite catalyst used in ex situ pyrolysis favored the production of aromatic hydrocarbons. The present study also showed that Zn/13X zeolite and Cr/13X zeolite catalysts achieved a conversion of the compounds containing oxygen over 97% in a combined process (in situ and ex situ pyrolysis variants), which is higher than either in situ or ex situ variant. Overall, this research study shows that the combined pyrolysis process using Zn/13X zeolite and Cr/13X zeolite catalysts and acid-pretreated biomass would be very effective in achieving a good conversion rate of the compounds containing oxygen to aliphatic and aromatic hydrocarbons and, therefore, to produce bio-oil of improved quality.

## Figures and Tables

**Table 1:** Amount of biomass and catalyst employed in the in situ, ex situ, or in the combined pyrolysis process.

Nanocatalyst	Abbreviation	Nanocatalyst (g)	Biomass (g)	Ratio C/B
In-situ/Ex-situ pyrolysis process				
13X zeolite	Ze-0.5	10	20	0.5
13X zeolite	Ze-1	20	20	1.0
13X zeolite	Ze-2	40	20	2.0
Zn/13X zeolite	ZnZe-0.5	10	20	0.5
Zn/13X zeolite	ZnZe-1	20	20	1.0
Zn/13X zeolite	ZnZe-2	40	20	2.0
Cr/13X zeolite	CrZe-0.5	10	20	0.5
Cr/13X zeolite	CrZe-1	20	20	1.0
Cr/13X zeolite	CrZe-2	40	20	2.0
Combined pyrolysis process (in-situ + ex-situ)				
Zn/13X zeolite (in situ pyrolysis)	ZnZe-2	40	20	2.0
Cr/13X zeolite (ex situ pyrolysis)	CrZe-2	40	20	2.0

**Table 2:** Proximate, ultimate, and component analysis of CCB and PTCCB samples.

Characteristics	CCB	PTCCB
Proximate analysis (wt.%, db)		
Moisture	8.27 ± 0.12	6.14 ± 0.25
Volatile matter	76.05 ± 1.42	80.52 ± 1.25
Fixed carbon	12.22 ± 0.27	11.19 ± 0.65
Ash content	3.46 ± 0.26	2.15 ± 0.35
Ultimate analysis (wt.%, db)		
Carbon	44.81 ± 0.24	48.96 ± 0.62
Hydrogen	5.83 ± 0.26	6.82 ± 0.15
Nitrogen	0.36 ± 003	0.42 ± 0.05
Sulfur	0.17 ± 0.01	0.26 ± 0.01
^a^ Oxygen	48.62 ± 0.27	43.19 ± 0.55
H/C molar ratio	1.56	1.67
O/C molar ratio	0.81	0.66
Component analysis (wt.%, db)		
Cellulose	31.74 ± 0.2	43.54 ± 0.2
Hemicellulose	32.42 ± 0.2	36.65 ± 0.2
Lignin	12.84 ± 0.2	16.76 ± 0.2
Empirical formula	CH_1.56_ O_0.81_ N_0.007_ S_0.001_;	CH_1.67_ O_0.66_ N_0.007_ S_0.002_
pH	5.46	5.06
^b^ GCVc (MJ/kg)	16.75	17.35

db—dry basis; ^a^ Calculated from difference; ^b^ Gross calorific value.

**Table 3:** The metals content in corn cob biomass (CCB) and pretreated corn cob biomass (PTCCB).

Metals Content (ppm)	Na	K	Ca	Mg	Fe	Al	Cu	Zn
CCB	55 ± 0.45	2720 ± 2.5	780 ± 10.5	3300 ± 3.45	980 ± 9.5	50 ± 0.5	60 ± 0.05	40 ± 0.3
PTCCB	10 ± 0.2	250 ± 0.2	12 ± 0.2	70 ± 5	47 ± 3.5	14 ± 0.2	3.65 ± 0.05	3 ± 0.05
Removal rate (%)	81.82	90.81	98.46	97.87	95.21	72.00	93.92	92.5

**Table 4:** 13X zeolite, Cr/13X zeolite and Zn/13X zeolite characteristics.

Material	S_BET_ (m^2^/g)	A_ps_ (nm)	V_tp_ (cm^3^/g)	S_MC_ (nm)
13X zeolite	682	6.12	0.678	-
Cr/13X zeolite	286	6.26	0.546	8.2
Zn/13X zeolite	218	6.85	0.507	28.5

Specific surface area (S_BET_); Average pore size (A_ps_); Total pore volume (V_tp_); Metal crystallite size (S_MC_).

**Table 5:** The acidity of 13X zeolite, Cr/13X zeolite and Zn/13X zeolite catalysts.

Material	Relative Acidity (μmol/g)			Temperature	Total Acidity (μmol/g)		
	a	b	c	(°C)	a	b	c
13X zeolite	26	-	35	125	-	750	62
Cr/13X zeolite	38	28	26	150	385	780	91
Zn/13X zeolite	-	41	30	-	250	685	72

**Table 6:** Main chemical compounds identified in bio-oil produced from combined pyrolysis process (in situ and ex situ) of corn cobs biomass, nanocatalyst-to-biomass ratio (C/B) was 2.

Compound	Formula	Retention Time (min)	Area%
2,4-methylhexane	C_8_H_18_	3.85	3.26
3-methylheptane	C_7_H_16_	3.88	1.51
1,4-dimethylcyclohexane	C_8_H_16_	3.94	5.03
Toluene	C_7_H_8_	4.31	0.68
2,6-dimethylheptane	C_9_H_20_	4.61	2.32
Furfural	C_5_H_4_O_2_	6.42	2.45
p-Xylene	C_8_H_10_	7.43	0.67
Styrene	C_8_H_8_	8.05	1.43
5-methyldecane	C_11_H_24_	8.36	4.83
Benzene,1,2-diethyl	C_10_H_14_	10.74	1.03
Phenol	C_6_H_6_O	10.81	12.92
Benzofuran	C_8_H_6_O	11.32	2.85
Indane	C_9_H_10_	12.82	7.85
2-methyl phenol	C_7_H_8_O	13.14	4.81
4-methyl phenol	C_7_H_8_O	13.82	7.62
Undecane	C_11_H_24_	14.51	1.12
2/3/4-ethyl phenol	C_8_H_10_O	14.79/16.55	2.19
2,3/4/6-dimethyl phenol	C_8_H_10_O	14.81/16.88	0.21
Benzene, hexyl	C_12_H_18_	15.51	0.67
3,4-dimethyl phenol	C_8_H_10_O	16.91	0.16
Naphthalene	C_10_H_8_	17.22	7.66
4-ethyl-2-methoxy phenol	C_9_H_12_O_2_	19.71	0.45
Naphthalene, 1-methyl	C_11_H_10_	20.38	3.78
Pentadecane	C_15_H_32_	20.56	0.81
Naphthalene, 2-methyl	C_11_H_10_	20.78	3.04
Biphenyl	C_12_H_10_	22.66	0.76
Naphthalene, 1-ethyl	C_12_H_12_	23.05	0.43
Acenaphthylene	C_12_H_8_	24.49	3.62
Acenaphthene	C_12_H_10_	25.33	0.74
Fluorene	C_13_H_10_	27.83	3.18
Anthracene	C_14_H_10_	32. 42	2.23
Phenanthrene	C_14_H_10_	32.71	1.04
2,6-Dimethoxy-phenol	C_8_H_10_O_3_	33.11	0.37
3-Methyl-1H-Indole	C_9_H_9_N	35.26	0.22
2,3,5-Trimethoxy toluene	C_10_H_14_O_3_	37.68	0.41
9-Octadecenoic acid, methyl ester	C_19_H_36_O_2_	50.05	0.03
Oleic acid	C_18_H_34_O_2_	51.84	0.26

GC-MS analysis; The values represent the average from two pyrolysis runs; Water content of 26.64 (wt.%) from bio-oil, determined by Karl Fischer titration.

## Data Availability

Not applicable.
